# Application of Chitosan and Its Derivative Polymers in Clinical Medicine and Agriculture

**DOI:** 10.3390/polym14050958

**Published:** 2022-02-28

**Authors:** Meng Zhang, Fengshi Zhang, Ci Li, Heng An, Teng Wan, Peixun Zhang

**Affiliations:** 1 Department of Orthopedics and Trauma, Peking University People’s Hospital, Beijing 100044, China; mengzh2008@bjmu.edu.cn (M.Z.); xmx066@pku.edu.cn (F.Z.); drlici@bjmu.edu.cn (C.L.); tengwan.med@hotmail.com (T.W.); 2 Key Laboratory of Trauma and Neural Regeneration, Peking University, Beijing 100044, China; 3 Beijing Key Laboratory for Bioengineering and Sensing Technology, Daxing Research Institute, School of Chemistry & Biological Engineering, University of Science & Technology Beijing, Beijing 100083, China; hengan@xs.ustb.edu.cn; 4 National Center for Trauma Medicine, Beijing 100044, China

**Keywords:** chitosan, chitosan derivative, biopolymers, biomedical, agriculture, delivery systems, wound dressing

## Abstract

Chitosan is a biodegradable natural polymer derived from the exoskeleton of crustaceans. Because of its biocompatibility and non-biotoxicity, chitosan is widely used in the fields of medicine and agriculture. With the latest technology and technological progress, different active functional groups can be connected by modification, surface modification, or other configurations with various physical, chemical, and biological properties. These changes can significantly expand the application range and efficacy of chitosan polymers. This paper reviews the different uses of chitosan, such as catheter bridging to repair nerve broken ends, making wound auxiliaries, as tissue engineering repair materials for bone or cartilage, or as carriers for a variety of drugs to expand the volume or slow-release and even show potential in the fight against COVID-19. In addition, it is also discussed that chitosan in agriculture can improve the growth of crops and can be used as an antioxidant coating because its natural antibacterial properties are used alone or in conjunction with a variety of endophytic bacteria and metal ions. Generally speaking, chitosan is a kind of polymer material with excellent development prospects in medicine and agriculture.

## 1. Introduction

**Figure 1:**
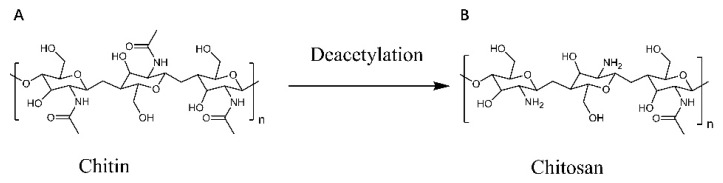
Preparation of chitosan by deacetylation. Chitosan (**B**) is formed by deacetylation of chitin (**A**).

Chitosan is a degradable multifunctional biopolymer obtained by chemical or enzymatic deacetylation from crustacean exoskeleton, insect cuticle, and fungal mycelium, which is a D-glucosamine linked by a β-1,4 glycosidic bond [1,2] (Figure 1A,B). By modifying chitosan, chitosan derivatives with different properties are generated, which are widely used in many fields such as biomedicine, agriculture, wastewater treatment, and food preservation [3,4]. In recent years, chitosan and its derivatives have received considerable attention for applications in nerve injury repair, wound repair, and as drug delivery systems due to their excellent properties in antibacterial, biocompatibility, and biodegradation [5].

The agricultural sector is facing great challenges with the increase in population, decrease in arable land, and increase in plant diseases caused by pathogens. Plant pathogens produce harmful toxins that cause slow crop growth and reduced yields, which can cause significant economic losses. However, over-reliance on existing fungicides to solve this problem is bound to have an impact on ecological diversity and public health safety. Therefore, there is an urgent need to develop antimicrobial drugs with new targets. Chitosan and its derivatives have become a research hotspot in the fields of plant growth stimulants and biological antimicrobial insecticides because of their broad-spectrum resistance to bacteria and fungi, biodegradability, biocompatibility, and plant growth-stimulating effects.

## 2. Physicochemical Properties of Chitosan

Chitosan has excellent biological properties such as biodegradability, biocompatibility, antibacterial, anti-tumor, hemostatic and antioxidant properties [6,7]. The biological properties of chitosan are closely related to its physicochemical structure, which includes the degree of deacetylation, water content, and molecular weight [8]. Deacetylation refers to the process of removing the acetyl group from chitosan, and this process determines the content of free amine groups in chitosan. Studies have shown that chitosan has good solubility only when the degree of deacetylation is above 85%. The enhanced chitosan uptake is mainly due to the interaction of positively charged chitosan with cell membranes, activation of chlorine–bicarbonate exchange channels, and reorganization of proteins associated with epithelial tight junctions, thus opening epithelial tight junctions [9,10]. Chitosan inhibits different species of microorganisms such as bacteria and fungi, and the mechanism is influenced by several factors, including the degree of deacetylation, pH, divalent cations, and solvent type. Oligomeric chitosan penetrates cell membranes more easily than high molecular weight chitosan and inhibits bacterial growth by inhibiting RNA transcription [8].

### 2.1. Chitosan Modification

Chitosan is widely found in nature and contains the following three functional groups: C2-NH2, C3-OH, and C6-OH as in Figure 1B. C3-OH has a large spatial site resistance and therefore is relatively difficult to modify. C2-NH2 is highly reactive for fine modifications and is the most common modifying group in chitosan [11]. In chitosan, although amino groups are more prone to nucleophilic reactions than hydroxyl groups, both can react non-selectively with electrophilic reagents such as acids, chlorides, and haloalkanes to functionalize them [12]. However, the low bioactivity and solubility of chitosan at physiological pH conditions due to its pH of about 6.5 certainly limits the application of chitosan. Therefore, reasonable modifications and modifications of chitosan are needed to obtain derivatives with increased activity and improved water solubility. Since chitosan contains a variety of functional groups, it can be functionalized in different ways such as phosphorylation, thiolation, and carboxylation (Table 1).

#### 2.1.1. Phosphorylated Chitosan

**Figure 2:**
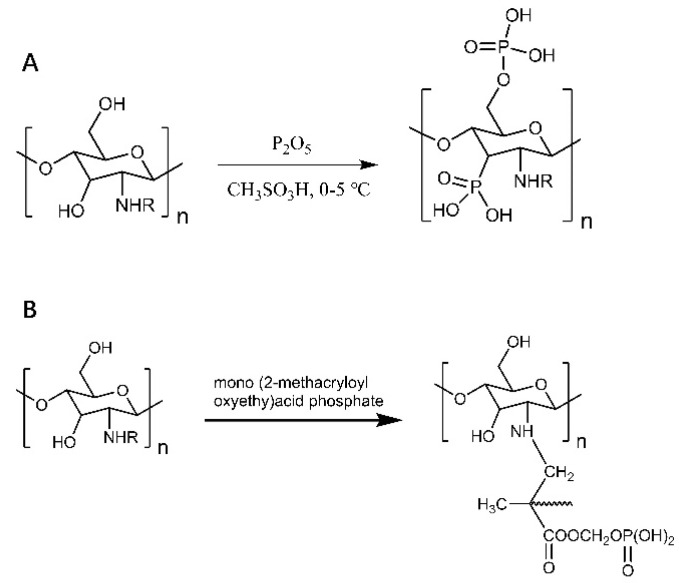
Phosphorylated chitosan. (**A**) Reaction of phosphorus pentoxide and chitosan to obtain highly substituted and water-soluble phosphorylated chitosan. (**B**) Preparation of phosphorylated chitosan by graft copolymerization of mono(2-methacryloyloxyethyl) phosphate chitosan.

The good water solubility and metal chelating properties of phosphorylated chitosan and its derivatives make them widely used in tissue engineering, drug delivery carriers, tissue regeneration, and the food industry [19,20,21]. As in Figure 2, water-soluble phosphorylated chitosan was obtained by the reaction of phosphorus pentoxide and chitosan under low-temperature conditions using methane sulfonic acid as the catalyst (Figure 2A); phosphorylated chitosan with good antibacterial activity and ionic properties was prepared by graft copolymerization of chitosan monophosphate [22,23] (Figure 2B). Phosphorylated chitosan has a better welling index and ionic conductivity properties. The crystallinity was reduced compared to chitosan, but the tensile strength was not significantly changed. Renata et al. improved the osteogenic activity of chitosan–xanthan gum scaffolds by introducing phosphate groups to chitosan’s amino and hydroxyl groups. In addition, signaling molecules, such as bone morphogenetic proteins and growth factors bound to the surface of phosphorylated chitosan, were able to induce directed differentiation of stem cells into osteoblasts [24]. Phosphorylated chitosan has a significant pro-value-added effect on osteoblasts, making it potentially valuable for a wide range of applications in bone tissue engineering. The solubility of chitosan-based hydrogels was significantly reduced at pH > 6.5. To solve this problem, the derivative N-methylene phosphonic acid chitosan (NMPC-GLU) was synthesized by cross-linking glutaraldehyde. The introduction of methylene phosphonic acid allows chitosan to improve solubility under pH-neutral conditions without reducing film-forming properties. Excellent compressive strength, elastic modulus, and promotion of cell proliferation were observed in the hydrogels prepared in this way [25].

#### 2.1.2. Thiolated Chitosan

Thiol chitosan is prepared by the derivatization of the amino group of chitosan with a thiol-containing coupling reagent [26,27]. The amino group at the 2nd position of chitosan glucosamine is the primary site of thiol group immobilization. The thiol-containing solvent attaches to chitosan by forming an amide bond with the primary amino group of chitosan. Under the conditions of amide bond formation, the carboxyl group of thioglycolic acid and the ligand cysteine mediate the reaction with the amino group of chitosan through water-soluble carbodiimide. The concentration of thiolate anions reflecting the activity of the thiol groups is low at pH < 5 during the reaction, which limits the formation of disulfide bonds [3]. The modified chitosan exhibits excellent properties due to the covalent attachment of the thiol groups to the chitosan. Additionally, the lower pH of the surrounding medium reduces the thiol chitosan activity, which reduces the oxidation of the modified chitosan and leads to enhanced adhesion properties [28,29,30]. Thiochitosan interacts with cysteines in membrane receptors, such as epidermal growth factor, causing intracellular tyrosine kinase activation, which enhances membrane permeability [31]. In conclusion, thiochitosan has good adhesion ability, inhibitory effect on phosphorylated glycoproteins, and enhanced membrane permeability. Li et al. used thiochitosan as a coating material to modify stainless steel containing maleimido-containing tannic acid (TAMA) and found a 70% reduction in adsorption to E. coli and a 90% reduction in adhesion [32,33]. The thiosemicarbazone coating modification method is important in inhibiting bacterial adhesion and biofilm formation on susceptible materials.

#### 2.1.3. Ionic Chitosan

**Figure 3:**
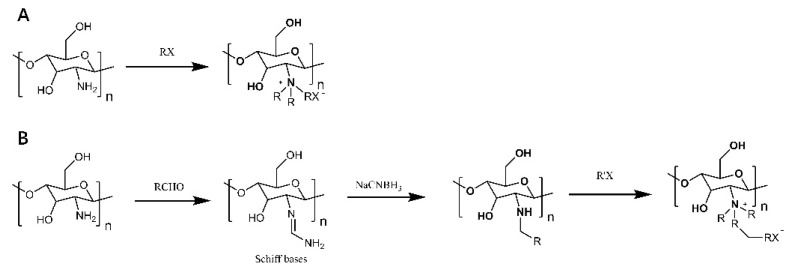
Direct quaternization of chitosan. (**A**) Processing of chitosan by haloalkanes under alkaline conditions. (**B**) Chitosan is first reacted with aldehyde, further reduced by sodium borohydride or sodium cyanoborohydride, and finally reacted with haloalkanes to obtain quaternized chitosan.

**Figure 4:**
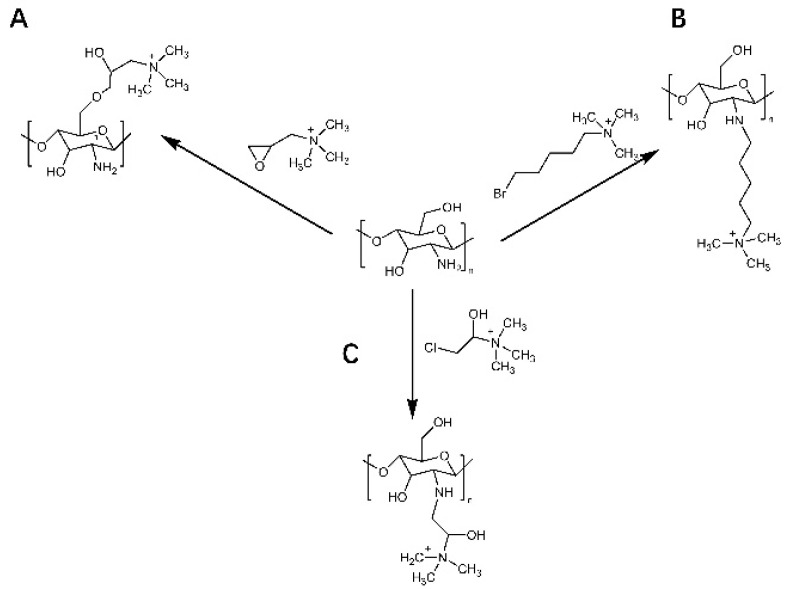
Indirect quaternization of chitosan. Introduction of small molecules containing quaternary ammonium groups into chitosan. (**A**) (5-bromopentyl)trimethylammonium bromide. (**B**) trimethylammonium chloride of glycidyl ester. (**C**) Preparation of quaternized chitosan by reaction of chitosan 3-chloro-2-hydroxypropyltrimethylammonium chloride.

Cationic derivatives of chitosan have important roles in bioadhesion, absorption enhancement, anti-inflammatory, antibacterial and anti-tumor applications. Chitosan modified with quaternary ammonium groups is one of the most common cationic chitosan derivatives. Quaternized chitosan with a permanent positive charge has increased antimicrobial activity and solubility compared to normal chitosan [34]. As illustrated in Figure 3, there are two main methods of chitosan quaternization: direct quaternization and indirect quaternization. One method of direct quaternization of chitosan amino acids is to treat chitosan with haloalkanes under alkaline conditions (Figure 3A). Another method is the reaction of chitosan with aldehydes first, further by sodium borohydride or reduction, and finally with haloalkanes to obtain quaternized chitosan [35,36] (Figure 3B). As seen in Figure 4, the indirect quaternization method refers to introducing small molecules containing quaternary ammonium groups into chitosan, such as glycidyl trimethyl ammonium chloride, (5-bromopentyl) trimethyl ammonium bromide, etc. [37,38] (Figure 4A,B). Tan et al. introduced quaternary ammonium groups into the chitosan backbone via azide-alkyne cycloaddition reactions to synthesize 1,2,3-triazole chitosan derivatives [39], which pointed the way to the antifungal and antioxidant properties of chitosan derivatives for a wide range of applications in biomedicine. Quaternized chitosan was prepared by dissolving chitosan in alkali and urea and then reacting it with 3-chloro-2-hydroxypropyl trimethylammonium chloride [40] (Figure 4C), which provides a simple and green solution to achieve chitosan functionalization.

## 3. Chitosan in Biomedical Applications

### 3.1. Anti-Tumor Effects of Chitosan and Its Derivatives

**Figure 5:**
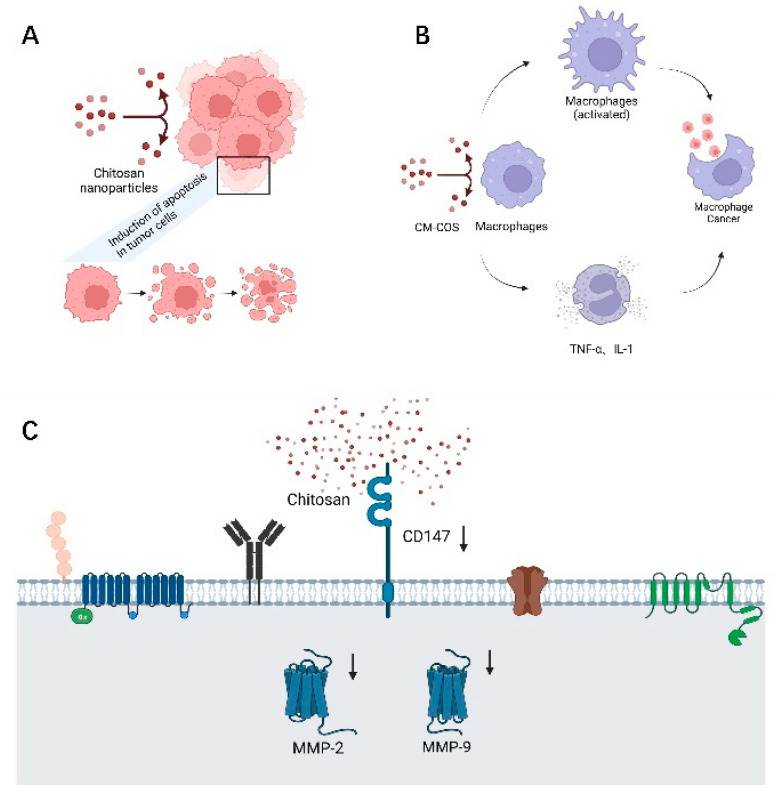
Anti-tumor effects of chitosan and its derivatives. (**A**) Chitosan induces apoptosis in tumor cells. (**B**) Carboxymethyl chitosan promotes the secretion of TNF-α and IL-1 cytokines and enhances macrophage phagocytosis. (**C**) Chitosan inhibits cancer cell metastasis by inhibiting MMP.

Cancer is one of the major diseases that threatens human health today, and according to statistics, it is responsible for about one-sixth of deaths worldwide [2]. Existing radiotherapy and chemotherapy usually destroy lymphocytes and bone marrow cells, causing serious side effects to the organism. Chitosan nanoparticles have a wide range of promising applications in anti-tumor effects, including hepatocellular carcinoma, gastric cancer, colorectal cancer, and lung cancer [5,41]. As indicated in Figure 5, chitosan has anti-proliferative activity against tumor cells, and Liu et al. showed that chitosan induced apoptosis in tumor cells by decreasing Bcl-2 and increasing Caspase-3 expression [42,43] (Figure 5A). Carboxymethyl chitosan (CM-COS) enhances macrophage viability, infiltrates heavily into the tumor microenvironment, produces cytokines such as TNF-α and IL-1, enhances phagocytosis and upregulates NO levels. Notably, CM-COS has inhibitory effects on sarcoma cells in vivo and on BEL-7402 cell growth in vitro but is not significantly toxic to normal liver cells [44,45] (Figure 5B).

In addition, chitosan has an inhibitory effect on tumor cell invasion and metastasis. Matrix metalloenzymes (MMP) are involved in the degradation of extracellular matrix during tumor invasion and metastasis, and MMP-2 can encode an enzyme that degrades type IV collagen [46]. Overexpression of MMP-2 is a prominent feature of gastric cancer, closely associated with gastric cancer metastasis. Chitosan can reduce the expression of cluster of differentiation 147 (CD147) and consequently MMP-2, thus showing a dose-dependent inhibition of metastasis of gastric cancer cells [47] (Figure 5C). Angiogenesis within tumor tissues may be related to the upregulation of MMP-9 expression leading to the release of vascular endothelial growth factor (VEGF). The formation of new blood vessels in tumor cells is one of the mechanisms of aggressive tumor metastasis, and VEGF and its receptors are regulatory signals for angiogenesis. Shen et al. found that VEGF expression was higher in lung metastases than in primary tumors [48].COS can inhibit VEGF expression by suppressing the expression of MMP-9, thus inhibiting tumor angiogenesis to suppress tumor invasion and metastasis [49].

### 3.2. Wound Healing

**Figure 6:**
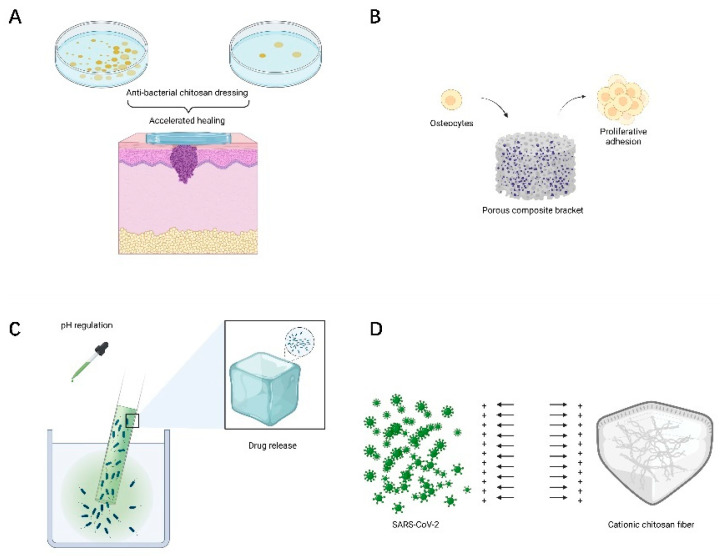
Other biomedical applications of chitosan. (**A**) Chitosan promotes wound healing. (**B**) Chitosan as a bone and cartilage tissue engineering repair material. (**C**) PH-sensitive chitosan drug delivery system. (**D**) The role of chitosan in the protection against SARS-CoV-2.

The ideal trauma dressing should provide a non-toxic, antibacterial, moist, and breathable microenvironment, thus promoting cell proliferation and accelerating wound healing. Positively charged chitosan can promote erythrocyte adhesion, fibrinogen adsorption, accelerate platelet aggregation and activation, and exhibit good hemostatic properties [50]. At the same time, chitosan presents good antibacterial activity due to its large amount of alkaline amino groups, which generally exhibit a cationic charge under acidic conditions, causing lysis and destruction of bacterial cells and protecting the wound surface from microbial infection [51]. Therefore, chitosan has good properties to promote wound healing and is an ideal material for the preparation of wound dressings. In addition, the morphological structure of chitosan nanofibers resembles the extracellular matrix of the skin, thus promoting wound healing [52] (Figure 6A). Zhang et al. prepared a nanocomposite hydrogel using aldehydic sodium alginate (SA), chitosan, and zinc oxide (ZnO) to enhance the antibacterial and coagulation properties [53]. It exhibited significant antibacterial activity against Staphylococcus aureus and Escherichia coli. Shen et al. prepared sulfated chitosan-doped I-sexual collagen hydrogels that enhanced macrophage polarization from M1 to M2. Stimulation of macrophage secretion of IL-4 and TGF-β1 enhanced collagen deposition, regenerative epithelialization, and neovascularization [54].

### 3.3. Tissue Engineering Materials for Bone and Cartilage

Tissue engineering is an emerging interdisciplinary discipline that combines material science, engineering mechanics, and biomedicine. The structure and function of damaged tissues and organs are repaired or replaced by cell transplantation combined with bioactive molecules and 3D scaffolds [55]. Mucopolysaccharides in the extracellular matrix play an important role in regulating chondrocyte morphology, function and differentiation. Chitosan is similar to mucopolysaccharides of the extracellular matrix and is used as a scaffolding material for cartilage tissue engineering [56]. The poor mechanical properties of chitosan were improved by synthesizing composites to improve the properties of hydrogels (Figure 6B). Kaviani et al. prepared nanoporous scaffolds using chitosan, collagen, and nanohydroxyapatite by the cryogel method, which reduced the rate of biodegradation and improved mechanical properties and facilitated cell proliferation and adhesion [3,57]. Porous scaffolds prepared by using chitosan, gelatin, and silk proteins have better compressive strength and compressive modulus, while incidental chondrocytes can form seed scaffolds and promote rapid regeneration of cartilage tissue [58]. Martino et al. prepared porous composite scaffolds by combining gelatin, chitosan, and polyvinyl alcohol with nano-hydroxyapatite [59]. The experimental results showed that the composite had good compression resistance, bionic structure, and high osteogenic differentiation ability. They effectively promoted the proliferation and adhesion ability of bone marrow mesenchymal stem cells. Therefore, the composite material is expected to be a bone repair and tissue engineering scaffold.

### 3.4. Nanocarriers for Drug Delivery

In recent years, nanomaterials for drug delivery systems have received a lot of attention in the pharmaceutical field. Chitosan is used as a cationic polysaccharide. Chitosan, as a cationic polysaccharide, contains a large number of amino and hydroxyl groups in its molecular chain, which makes it PH sensitive [60]. The design of such PH stimuli-responsive nanohydrogels provides a new idea for drug slow release and delivery. Lactic acid-modified carboxymethyl chitosan methacrylate was cross-linked with ortho-methacrylate monomer to prepare pH-sensitive, tumor-targeting bifunctional nanogels, which improved the growth inhibition and penetration of tumor cells in vitro [61]. This pH-induced drug-release behavior improved its tumor-targeting ability and is expected to be a tumor drug delivery vehicle (Figure 6C).

Cationic chitosan can bind to the negative charge of DNA through ionic bonds to form stable multimers. At the same time, electrostatic interactions between chitosan and genes can prevent DNA from degradation by nucleases [62,63]. This makes chitosan a promising gene carrier. Delivery of small DNA and short interfering RNA (siRNA) is an important tool to inhibit oncogene expression. siRNA and chitosan form chitosan nanoparticles Chitosan-Based Nanoparticles (CS-NPs). It protects siRNA from enzymatic breakdown and translocates siRNA into target cells [62,64].

It was shown that the longer the blood circulation time of chitosan kinase particles, the more efficient their penetration and retention in the organism. This also suggests that the particle size, particle shape, and surface charge of nanoparticles also affect their blood circulation time and membrane penetration efficiency. Ultimately, to effectively achieve the penetration effect of chitosan, NPs should be able to remain stably in the bloodstream and thus have a better chance to penetrate the tumor vasculature and accumulate in the tumor. Table 2 demonstrates the chitosan-based nanocarriers and their mechanism of action.

### 3.5. Potential Application of Chitosan in the Fight against COVID-19

The endemic strain of coronavirus SARS-CoV-2 is the main causative agent of the novel coronavirus pneumonia (COVID-19). Chitosan can combat viral infections by direct antiviral activity and by inducing antiviral immune response mechanisms. Cationic chitosan can kill viruses by interacting with their negative charges through electrostatic interactions to disrupt their protective membranes [72]. N-(2-hydroxypropyl)-3-methylchitosan ammonium chloride has a strong interaction with the viral S protein, thus forming a complex to prevent viral entry into host cells [73]. Chitosan enhances its antiviral immune response by stimulating immune cells, activating alternative pathways of the complement system [74]. Chitosan is phagocytosed by macrophages and promotes the production of reactive oxygen species (ROS). ROS induces IFN synthesis by splenocytes to exert antiviral effects by inhibiting the translation of viral mRNA [75].

SARS-CoV-2 is mainly transmitted through the respiratory tract or droplets. Infected patients produce aerosol particles by breathing, and the particles are too small to settle, which increases the risk of exposure to health care workers [76]. Hathout et al. proposed the use of positively charged polymers such as chitosan to prepare nanofibers with large surface areas and embed them in protective clothing. The electrostatic repulsion between chitosan nanofibers and positively charged SARS-CoV-2 could reduce the viral load around the protective clothing, thus reducing the ability of the virus to spread [77] (Figure 6D). Chitosan nanoparticle aerosols made by Bioavanta can deliver any potentially anti-COVID-19 drug to the lungs. The nanoparticles adhere to the airways and the encapsulated active substance is released due to slow degradation, thus ensuring a high local concentration of the drug [75]. Therefore, chitosan delivery of drugs via the intranasal mucosal route is a very promising approach against SARS-CoV-2.

## 4. Application of Chitosan in the Field of Agriculture

### 4.1. Plant Growth Stimulants

**Figure 7:**
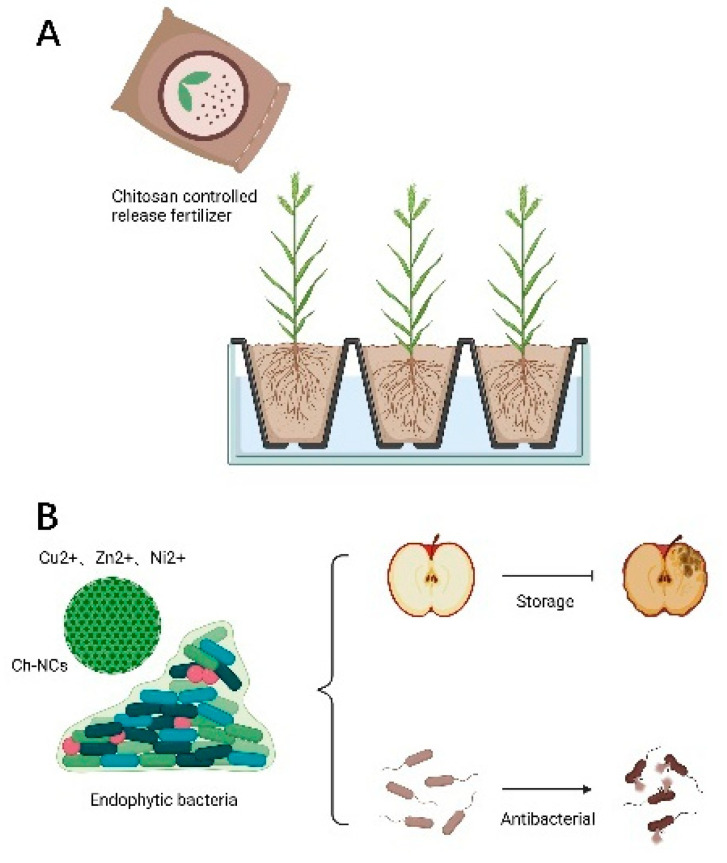
Application of chitosan in the field of agriculture. (**A**) Chitosan can be used to prepare stimulation-controlled drug release systems. (**B**) Chitosan synergizes with metal or endophytic bacteria to achieve antiseptic and inhibit harmful bacteria.

Fertilizers, pesticides, and large amounts of nutrients are currently the main ways to promote plant growth and development and increase crop yields. However, the continuous use of pesticides is bound to affect the sustainable development of the agricultural economy. In this era of precision agriculture, new environmentally-friendly nano-fertilizers have received great attention. The biodegradability of chitosan can be used to prepare stimulation-controlled drug release systems. Chitosan nanocarriers respond positively to small environmental signals such as ambient temperature and light. It causes changes in structure, solubility and transforms into a gel, which facilitates release. Once the stimulation stops, it returns to the initial structure [78,79]. Stimulation-controlled chitosan nanofertilizers can improve utilization, reduce the dose used, and effectively promote plant growth. Chitosan improves plant tolerance to salinity, high temperature, and drought, and increases photosynthetic efficiency [80]. The hydrophilic nature of chitosan reduces transpiration and promotes water uptake [81,82]. As depicted in Figure 7, chitosan can be used as an additional carbon source in plant synthesis to promote plant growth [83] (Figure 7A). Saharan et al. found that seeds treated with 0.04% and 0.12% Cu-chitosan nanoparticles (NPs) had higher germination, seedling length, root length, and root number than the control group [84]. Cu-chitosan NPs can increase protease and α-amylase activities to promote the mobilization of protein and starch to promote seedling growth.

### 4.2. Chitosan-Based Nanocomplexes (Ch-NCs) for Fungicides

Chitosan has an inhibitory effect on fungi and bacteria and is a good antimicrobial drug. Cationic Ch-NCs bind to negatively charged microbial cell membranes, leading to changes in cell membrane permeability and leakage of cellular components, causing cell death. Ch-NCs interfere with mRNA and protein synthesis by penetrating into the nucleus of microbial cells and binding to DNA. It can also selectively bind to metals, thereby inhibiting the production of microbial toxins. Alternatively, it inhibits oxygen and nutrient uptake by forming a blocking layer on the microbial cell wall [85,86,87,88]. It was found that quaternary chitosan has antibacterial activity against Escherichia coli and Staphylococcus aureus, and the antibacterial activity increased with the increasing chain length of alkyl substituents. Ch-NCs can significantly inhibit the growth of Ch-NCs, which is essential for eradicating and preventing bacterial diseases in crops. In addition, Ch-NCs are used in soil conditioners, foliar, and seed treatments. It can inhibit the growth of fungi such as gray mold fungus and Aspergillus flavus at different growth stages, such as mycelial growth, sporulation, and fungal toxin factor production [89]. It was found that injection of spore suspensions of A. gray mold on strawberry fruits, coated with chitosan, and stored at 13 °C for 14 days significantly slowed down fruit decay (Figure 7B). Therefore, Ch-NCs have a vast application potential as edible antifungal coating material after fruit picking. ε-polylysine and chitosan combination fungicides inhibited tomato gray mold fungus up to 90% [90]. Cobos et al. found a significant reduction in infection and mortality of fungal pathogens of grapevine trunks after treatment with chitosan in experimental fields [91].

### 4.3. Synergistic Effects of Chitosan and Metals

Metals such as zinc, copper, silver, and nickel are highly compatible with chitosan NPs and are harmless to plant systems. Cu2+, Zn2+, and Ni2+ have fungicidal and disinfectant properties, and when chitosan is combined with metal ions, it enhances the antibacterial activity against A. solanacearum, A. griseus, and A. curvatus. Abd-Elsalam et al. used metal organosols to impregnate chitosan to prepare Ch-Zn NCs, the nanomaterials exhibited good antifungal activity and even inhibited the growth of A. solanacearum [92]. Zn is a cofactor of enzymes involved in protein metabolism and replication of DNA and plays a vital role in the plant immune system. Ch-Zn NCs enhance plant immune response by increasing defense enzymes and antioxidant activity [93]. The formation of reactive oxygen species (ROS) is dependent on light, and after ROS production, they can be rapidly eliminated by antioxidant enzymes. Chitosan can induce plant chloroplasts (or mitochondria) to produce H2O2, which is involved in the scavenging of reactive oxygen species. Chitosan NPs can also increase the levels of antioxidant enzymes such as peroxidase (POD), catalase (CAT), superoxide dismutase (SOD), and glutathione reductase (GR). Ch-Ag NPs have POD activity and catalyze the breakdown of H2O2 into hydroxyl radicals, which facilitates plants against unfavorable environments [94,95,96].

### 4.4. Synergistic Effect of Chitosan and Endophytic Strains

Endophytic bacteria are microorganisms that colonize the inside of plants at some or all stages of their life history. Endophytic bacteria can produce phytohormones and growth regulators that increase photosynthetic activity and promote plant growth. Drought stress is usually accompanied by ROS formation, which can damage macromolecules and cell membranes. Endophytic bacteria can alter the activity of antioxidant enzymes associated with drought stress to increase drought tolerance in wheat. Endophytic bacteria and chitosan treatment help plants to adapt to drought, scavenge ROS toxic compounds, and reduce cellular damage [97,98]. Endophytic bacteria, such as B. cepacian, Bacillus spp., and Pseudomonas, can break down deaminase precursors and reduce ethylene concentration in plant tissues, while the end products are carbon and nitrogen sources required for plant growth and promote plant growth under abiotic stress conditions [99,100]. Kumar et al. found that treatment when Pseudomonas and chitosan were applied together, sorghum seedlings grew faster. Thus, the combination of chitosan with endophytic bacteria, such as Bacillus cereus, promoted plant growth and control of biological diseases caused by pathogenic bacteria [101].

## 5. Conclusions and Future Prospects

Chitosan, as a biopolymer obtained by deacetylation, is widely appreciated for its biocompatibility, degradability, antimicrobial properties, and modifiability. Its derivatives can also obtain superior physicochemical properties. This has led to a wider range of applications. The research results of scientists from various countries have demonstrated that chitosan is not only limited to the biomedical field, aiding in solving complex problems nowadays in skin damage, orthopedic endophytes, and even virus protection, but also has applications in the agricultural field.

This paper presents a review of the preparative modification of chitosan and its recent applications in biomedical and agricultural fields. The functional groups and interactions that play an essential role in the modification of chitosan are systematically explained. The functional groups with specific functions are introduced differently due to the modifications (e.g., phosphorylation, sulfhydration, cationization, etc.), thus making them controllable. The wide variety of structural properties and application directions of chitosan derivatives is particularly advantageous to ensure their wide application. Carboxymethyl chitosan exhibits anti-tumor effects by promoting autophagy of tumor cells and enhancing the secretion and phagocytic activity of macrophages. The amino group contained in chitosan has an antimicrobial function and offers the possibility of wound dressing improvement. Complex porous scaffolds made of chitosan have improved mechanical properties and have been progressively applied to bone endosseous plants to promote the crawling and growing of bone cells. In addition, PH-sensitive chitosan can offer the possibility of controlled drug release. Surprisingly, the positive charge carried by cationic chitosan has potential applications in protective gear urgently needed for novel coronary diseases. In agriculture, specific condition-sensitive chitosan can assist in the sustained release of plant nutrient drugs to achieve controlled slow release, plant growth promoters, and fungicidal effects. It is also able to act synergistically with metal ions and plant endophytic bacteria to promote plant growth. In the future, pre-engineered chitosan derivatives with good biocompatibility will have promising applications in biomedical and agricultural fields.

## Figures and Tables

**Table 1:** Modification of chitosan and its derivatives and their significant properties.

Types	Functional Group	Common Derivatives	Applications	Reference
Thiolated chitosan derivatives	the thiol groups	chitosan-6-mercaptonicotinic acid; chitosan-4-thiobutylamidine;	Tissue-engineered scaffolds; Drug delivery nanocarriers; Wastewater treatment; Antibacterial activity;	[13]
Phosphorylation of chitosan derivatives	phosphate groups	N-methylene phosphonic chitosan	Induced bone formation; Increased solubility; Fuel cell;	[14]
Crosslinked chitosan derivatives	cross-linking bridges	Ethylene diamine tetraacetic acid chitosan polymer; Chitosan-glutaraldehyde cross-linked polymers;	Drug delivery systems; Biosorbents;	[15]
Carboxylic acid chitosan derivatives	Carboxymethyl group	N, O-carboxymethyl chitosan; N, N-carboxymethyl chitosan; Chitosan-methacrylate;	Tissue engineering scaffold materials; food industry; nanobiosensors	[16]
Cationic chitosan derivatives	quaternary functional groups	N-(2-hydroxy) propyl-3-trimethylammonium chitosan; chitosan-(mono, di, tri)chloroacetate; chitosan-trifluoroacetate;	Antibacterial activity; Increased water solubility; Antioxidant reagent;	[17]
Sulfated chitosan derivatives	–SO_4_^2−^ groups	6-O-sulfated chitosan; 2-N,6-O-sulfated chitosan;	Tissue repair and regeneration; anticoagulation; antioxidant; antiviral	[18]

**Table 2:** Chitosan-based nanocarriers and their mechanism.

Nanocarrier Component	Loaded Drug	Vivo or Vitro	Efficacy Mechanism	Reference
Chitosan nanocarrier	Gemcitabine	In vitro	Oral adsorption	[65]
Chitosan/poly(ethylene glycol)	Gemcitabine	In vitro and in vivo	Reduce the burden of frequent dosing and higher toxicity	[66]
Chitosan nanoparticle	Herceptin (HER2)conjugated gemcitabine	In vitro	Eventual uptake and prolonged intracellular retention	[67]
O-carboxymethyl chitosan	Curcumin	In vitro	Increase drug solubility	[68]
N-octyl-O-sulfate chitosan micelles	Paclitaxel	In vitro	Solubilization of hydrophobic drugs	[69]
Glycol chitosan–5βcholanic acid (HGC)	Camptothecin	In vitro and in vivo	Increase drug stability, solubility and retention	[70]
Hydroxyapatite chitosan nanocomposite	Celecoxib	In vitro and in vivo	Sustained-release patterns	[71]

## Data Availability

The data presented in this study are available on request from the corresponding author.
